# Inhibition of 5-Lipoxygenase Pathway Attenuates Acute Liver Failure by Inhibiting Macrophage Activation

**DOI:** 10.1155/2014/697560

**Published:** 2014-06-01

**Authors:** Lu Li, Yi-Rong Liu, Shan Gao, Jun-Feng Li, Shan-Shan Li, Dan-Dan Zhang, Shuang Liu, Li Bai, Su-Jun Zheng, Zhong-Ping Duan, Min Qi, Yu Chen

**Affiliations:** ^1^Artificial Liver Center, Beijing YouAn Hospital, Capital Medical University, Beijing 100069, China; ^2^Department of Toxic Hepatic Diseases, Beijing YouAn Hospital, Capital Medical University, Beijing 100069, China; ^3^Department of General Medicine, Luoyang Central Hospital, Zhengzhou University, Luoyang 471000, China

## Abstract

This study aimed to investigate the role of 5-lipoxygenase (5-LO) in acute liver failure (ALF) and changes in macrophage activation by blocking it. ALF was induced in rats by administration of D-galactosamine (D-GalN)/lipopolysaccharide (LPS). Rats were injected intraperitoneally with AA-861 (a specific 5-LO inhibitor), 24 hr before D-GalN/LPS administration. After D-GalN/LPS injection, the liver tissue was collected for assessment of histology, macrophage microstructure, macrophage counts, 5-LO mRNA formation, protein expression, and concentration of leukotrienes. Serum was collected for detecting alanine aminotransferase (ALT), aspartate transaminase (AST), total bilirubin (Tbil), and tumor necrosis factor- (TNF-)***α***. Twenty-four hours after injection, compared with controls, ALF rats were characterized by widespread hepatocyte necrosis and elevated ALT, AST, and Tbil, and 5-LO protein expression reached a peak. Liver leukotriene B4 was also significantly elevated. However, 5-LO mRNA reached a peak 8 hr after D-GalN/LPS injection. Simultaneously, the microstructure of macrophages was changed most significantly and macrophages counts were increased significantly. Moreover, serum TNF-***α*** was also elevated. By contrast, AA-861 pretreatment significantly decreased liver necrosis as well as all of the parameters compared with the rats without pretreatment. Macrophages, via the 5-LO pathway, play a critical role in ALF, and 5-LO inhibitor significantly alleviates ALF, possibly related to macrophage inhibition.

## 1. Introduction

Acute liver failure (ALF), characterized by massive necrosis, is a syndrome defined by the onset of coagulopathy and hepatic encephalopathy and has a mortality rate ranging from 50% to 70% [1–6]. The prognosis of this disease has not significantly improved, despite the introduction of supporting treatments such as plasma exchange, dialysis, and antibiotics [7–10]. Although liver transplantation is an effective treatment, it is not universally applicable. With the limited understanding of the pathogenesis, no effective therapy is available to prevent or treat this disease.

Recently, several authors have reported the increased serum levels of macrophage­derived factors in patients with ALF, irrespective of the trigger, which suggests that activated macrophages play an important role in the progression of ALF [11–13]. Indeed, activation of macrophages and the subsequent release of proinflammatory mediators such as cytokines (e.g., tumor necrosis factor (TNF)-*α*), reactive oxygen species (ROS), and eicosanoids are considered to be an early step in the pathogenesis of liver damage, because they stimulate hepatic inflammation. Moreover, in the animal model of liver disease, the number of macrophages increases consistently and correlates closely with the degree of hepatic injury [14]. Thus, selective inactivation of macrophages might be a potential approach for the treatment of ALF.

In the liver, macrophages are the only cell type endowed with a metabolically active 5-lipoxygenase (5-LO) pathway (i.e., 5-LO, 5-LO-activating protein (FLAP), leukotriene (LT) C4 synthase, and generated LTB4 and cysteinyl-LTs), which is necessary for the biosynthesis of LTs [15]. LTs are metabolites of the 5-LO pathway and have been shown to enhance injury to organs during endotoxemia. In addition, LTB4 is a chemoattractant for leukocytes and plays a pivotal role in the pathogenesis of inflammatory and immune diseases [16]. Thus, the 5-LO pathway leading to the production of LTs is a major proinflammatory pathway in macrophages.

The expression of 5-LO in the liver is restricted to macrophages and 5-LO is necessary for the progression of ALF. We hypothesize that the mechanism of blocking the 5-LO pathway protects the liver from necroinflammatory damage and may be related to inhibiting activation of macrophages. Therefore, 5-LO inhibitors may have a therapeutic effect on ALF by inhibiting macrophage activation. Using AA-861 as a specific 5-LO inhibitor, the present study investigated the role of 5-LO in ALF in rats and changes in activation of macrophages by blocking it.

## 2. Materials and Methods

### 2.1. Experimental Animals

Male Wistar rats (weighing 150–180 g) were obtained from the Academy of Military Medical Sciences, China. They were acclimatized to animal house conditions and were fed a standard pellet diet and water* ad libitum* for 1 week. The experiments were conducted according to the ethical norms approved by the Ministry of Social Justices and Empowerment, China, and Institutional Animal Ethics Committee guidelines. This study was also approved by Ethics Committee of Beijing YouAn Hospital (Review number 2012-58).

### 2.2. Main Chemicals

D-Galactosamine (D-GalN), lipopolysaccharide (LPS), AA-861, dimethyl sulfoxide (DMSO), Tween, and phenylmethylsulfonyl fluoride (PMSF) were purchased from Sigma (St. Louis, MO, USA). Dulbecco-Vogt phosphate-buffered saline (DPBS), LTB4 Elisa kit, and Sep-Pak C18 cartridges were obtained from Cayman Chemical Co. (Ann, Arbor, MI, USA). *β*-Mercaptoethanol, tetramethylethylenediamine (TEMED), and diethypyrocarbonate (DEPC) were purchased from Amresco Biochemical Co. ( Solon, OH, USA). RIPA and TRIzol RNA were purchased from Invitrogen (Carlsbad, CA, USA). The microbicinchoninic acid (BCA) protein assay reagent kit was from Pierce (Rockford, IL, USA). Immobilon-P transfer membranes were from Millipore (Bedford, MA, USA). Mouse anti-rat monoclonal ED1 antibody and rabbit anti-rat polyclonal 5-LO antibody were provided by Abcam (Cambridge, MA, USA). Rabbit anti-rat monoclonal *β*-actin antibody was purchased from Epitomics (Burlingame, CA, USA). TNF-*α* Elisa kit was provided by IBL (Minneapolis, MN, USA). The SuperScript II Reverse Transcriptase Kit, TaqMan Gene Expression Assay, and universal TaqMan 2x polymerase chain reaction (PCR) master mix were from Takara Bio (Dalian, China).

### 2.3. Animal Model

Seventy-six male Wistar rats were randomly divided into three groups. (1) Control group (6 rats): DMSO (1.5 mL) was injected intraperitoneally. After 12 hr, the rats were fasted for a further 12 hr and 0.9% NaCl (2 mL) was injected intraperitoneally. Twenty-four hours after saline injection, the rats were killed and the liver tissues and serum were collected for further analysis. (2) ALF group (35 rats): DMSO (1.5 mL) was injected intraperitoneally. After 12 hr, the rats were fasted for a further 12 hr and D-GalN/LPS (1200 mg/kg D-GalN, 100 *μ*g/kg LPS) was injected intraperitoneally. Six rats were killed randomly 8 and 24 hr, respectively, after D-GalN/LPS administration and the liver tissue and serum were collected for further analysis. (3) AA-861 prophylactic group (35 rats): AA-861 (60 mg/kg) was injected intraperitoneally 12 hr later, the rats were fasted for a further 12 hr, and D-GalN/LPS (1200 mg/kg D-GalN, 100 *μ*g/kg LPS) was administrated intraperitoneally. Six rats were killed randomly after 8 and 24 hr, respectively, after D-GalN/LPS administration and the liver tissue and serum were collected for further analysis. Twenty rats in groups 2 and 3 were housed for further survival observation.

### 2.4. Biological Assays

Serum alanine aminotransferase (ALT), aspartate aminotransferase (AST), and total bilirubin (Tbil) levels were measured with an autoanalyzer (AU 5400; Olympus Optical Co., Japan).

### 2.5. Histological Analysis

Liver samples were cut into 5-mm sections, fixed in 4% formaldehyde and embedded in paraffin, and stained with hematoxylin and eosin for histological analysis. Hepatocellular necrosis was assessed by a registered pathologist who was unaware of the treatments. Parts of Modified Histological Activity Index were performed to evaluate the necroinflammatory degree (confluent necrosis score and spotty lytic necrosis, apoptosis, and focal inflammation score).

### 2.6. Transmission Electron Microscopy of Macrophages

Liver samples were cut into 1-mm sections and fixed in 2.5% glutaraldehyde with 2% paraformaldehyde for 2 hr at 4°C. The liver samples were washed three times for 10 min each with sodium dimethyl arsenic acid buffer (pH 7.2) and fixed in 1% osmic acid for 2 hr at 4°C. Two hours later, tissues were washed three times for 10 min each with double-distilled water. The samples were dehydrated through an ethanol gradient (50%, 70%, and 90%, 10 min each), anhydrous alcohol, and epoxy propane twice for 15 min each. After the metathesis step, liver samples were embedded in pure phenolic resin. Polymerization conditions were 30°C (5 hr), 50°C (5 hr), and 80°C (5 hr). Semithin sections were made and dyed with azure-meilan and positioned under light microscope. Ultrathin sections were cut and stained with uranyl acetate and lead citrate. The sections were observed under a transmission electron microscope (H-7650, Hitachi, Japan).

### 2.7. 5-LO  and Macrophages  Quantified  by Immunohistochemistry

ED1-positive or 5-LO-positive cells were detected immunohistochemically by the avidin-biotin complex method using a Vectastain elite ABC kit as described previously [17], with modifications. Liver sections were deparaffinized and incubated with preheated (37°C) 0.1% trypsin solution for 15 min and then with 3% H_2_O_2_ for 10 min to quench endogenous peroxidase. To block nonspecific reactions, sections were incubated with goat blocking serum for 30 min and then with avidin-biotin blocking solution according to the manufacturer's instructions. Following blocking, sections were incubated overnight at 4°C with mouse anti-rat monoclonal ED1 antibody (1 : 150) or rabbit anti-rat polyclonal 5-LO antibody (1 : 100) as the primary antibody and then incubated for 30 min with a biotinylated goat anti-mouse antibody or a biotinylated goat anti-rabbit antibody. Sections were incubated for 30 min with the avidin-horseradish peroxidase (HRP) complex, then treated with DAB (3,3N-Diaminobenzidine Tetrahydrochloride) Horseradish Peroxidase Color Development Kit for 5 min, and finally counterstained with hematoxylin. Sections were dehydrated, cleared, and mounted in VectaMount mounting medium. For quantitative analysis, the number of ED1-positive or 5-LO-positive cells was counted in a total of 25 high-power fields (HPFs) per tissue section under a light microscope (BH-2; Olympus Optical Co., Japan) at 100, 200, and 400x magnification, and results were presented as number of positive cells/HPF.

### 2.8. LTB4 in Liver Tissue and TNF-*α* in Serum

Liver tissue (120 mg) was homogenized with an Ultra-Turrax basic homogenizer (Biospec, Bartlesville, OK, USA) in 4 mL DPBS and kept with 2 vol. ice-cold methanol. Homogenates were centrifuged at 2000 rpm for 10 min at 4°C, and eicosanoids present in the supernatants were extracted with Sep-Pak C18 cartridges. The eluted methyl formate and methanol fractions were taken for further analysis of LTB4 levels, by specific enzyme immunoassay kit [14].

Blood collected from the inferior vena cava was centrifuged at 1500 rpm for 10 min, and the supernatant was used for TNF-*α* analysis.

### 2.9. 5-LO mRNA Transcription

Total RNA (2.5 *μ*g) was isolated from rat liver tissue and reverse-transcribed into cDNA with the SuperScript III First-Strand Synthesis System (Invitrogen). The RT reaction mixture (in a final reaction volume of 20 *μ*L) contained the following: 10 *μ*L 1x SuperMix (SYBR Premix Ex Taq; Takara), 2 *μ*L cDNA, 0.4 *μ*L 50x Rox Reference Dye I, 6.8 *μ*L dH_2_O_2_, and 0.4 *μ*L each primer. Real-time quantitative PCR was performed with an ABI Prism 7900 sequence detection system (Applied Biosystems, Foster City, CA, USA). 5-LO primer sequence is as follows: forward: 5′-ACGTTTATGGCATGCGGGGC-3′; reverse: 5′-ATTGCGCTCGGCAATCACGC-3′, using *β*-actin as an endogenous control. Amplification conditions were 50°C for 2 min and 95°C for 10 min, followed by 40 cycles of 95°C for 15 s and 60°C for 1 min. PCR results were analyzed with the sequence detector software version 2.1 (Applied Biosystems). Relative quantitation of gene expression was calculated using the standard curve method.

### 2.10. Total 5-LO Protein Quantification by Western Blotting

Liver tissue stored at –80°C was homogenized in 8 vol. 10 mM HEPES/KOH, pH 7.4, containing 0.25% (w/v) sucrose, 2 mM EDTA, aprotinin (1 *μ*g/mL), leupeptin (1 *μ*g/mL), pepstatin A (1 *μ*g/mL), and 10 *μ*M PMSF. After centrifugation at 1200 rpm for 15 min at 4°C, the supernatant was collected and the protein concentration was measured spectrophotometrically using the BCA method (protein assay) following the Pierce Co.'s protocol. Western blot analysis for 5-LO was performed as described previously [18]. Total protein (160 *μ*g) was subjected to 12% SDS-PAGE and transferred to PVDF nitrocellulose membranes. Antibodies against phosphorylated and total 5-LO, as well as *β*-actin, were used for western blot analysis. Membranes were probed with primary antibody (1 : 750) in 10 mL blocking buffer overnight at 4°C. After washing, membranes were further probed with appropriate HRP-conjugated secondary antibody (1 : 3000) in 10 mL blocking buffer for 1 h at room temperature. SuperSignal West Pico Chemiluminescent Substrates from Bio-Rad ChemiDoc MP (Shanghai, PRC) were used for chemiluminescence development.

### 2.11. Statistical Analysis

Statistical analysis was performed by analysis of variance (one-way or two-way) or unpaired Student's *t*-test. Results are expressed as the mean ± standard error of the mean (SEM). Differences were considered significant at *P* < 0.05 (two-tailed).

## 3. Results

**Figure 1:**
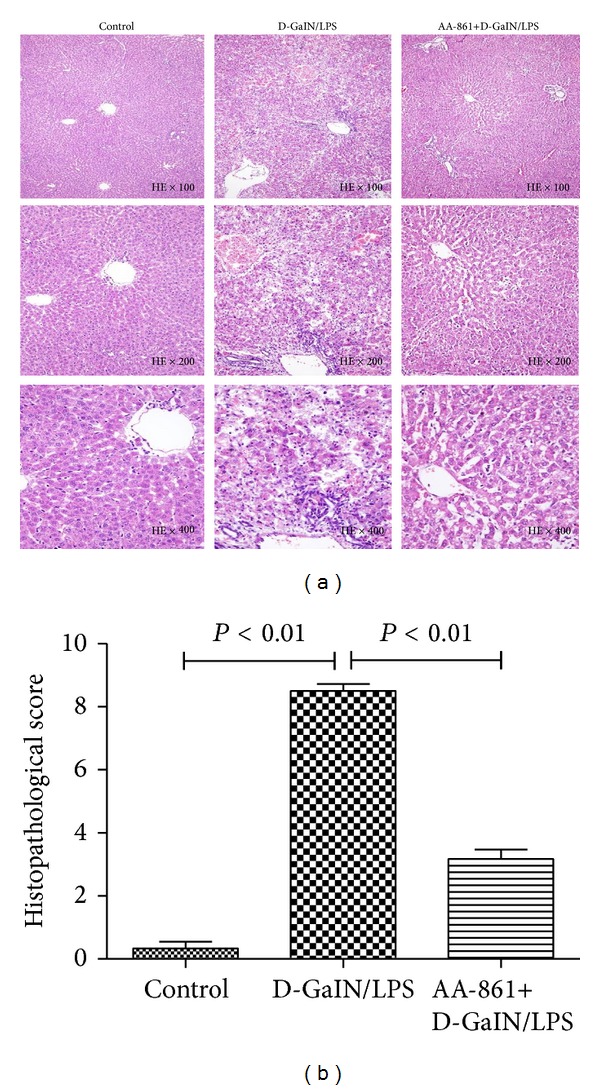
5-LO specific inhibitor AA-861 prevented hepatocellular necrosis in D-GalN/LPS-treated rats. Representative photomicrographs of liver sections stained with hematoxylin and eosin from the control group receiving placebo (left panels), 24 hr D-GalN/LPS-treated rats receiving placebo (middle panels), and 24 hr D-GalN/LPS-treated rats receiving AA-861 (right panels). Confluent necrosis score and spotty lytic necrosis, apoptosis, and focal inflammation score in Modified Histological Activity Index system were employed. Control: 0; D-GalN/LPS: 9; AA-861 + D-GalN/LPS: 2.

**Figure 2:**
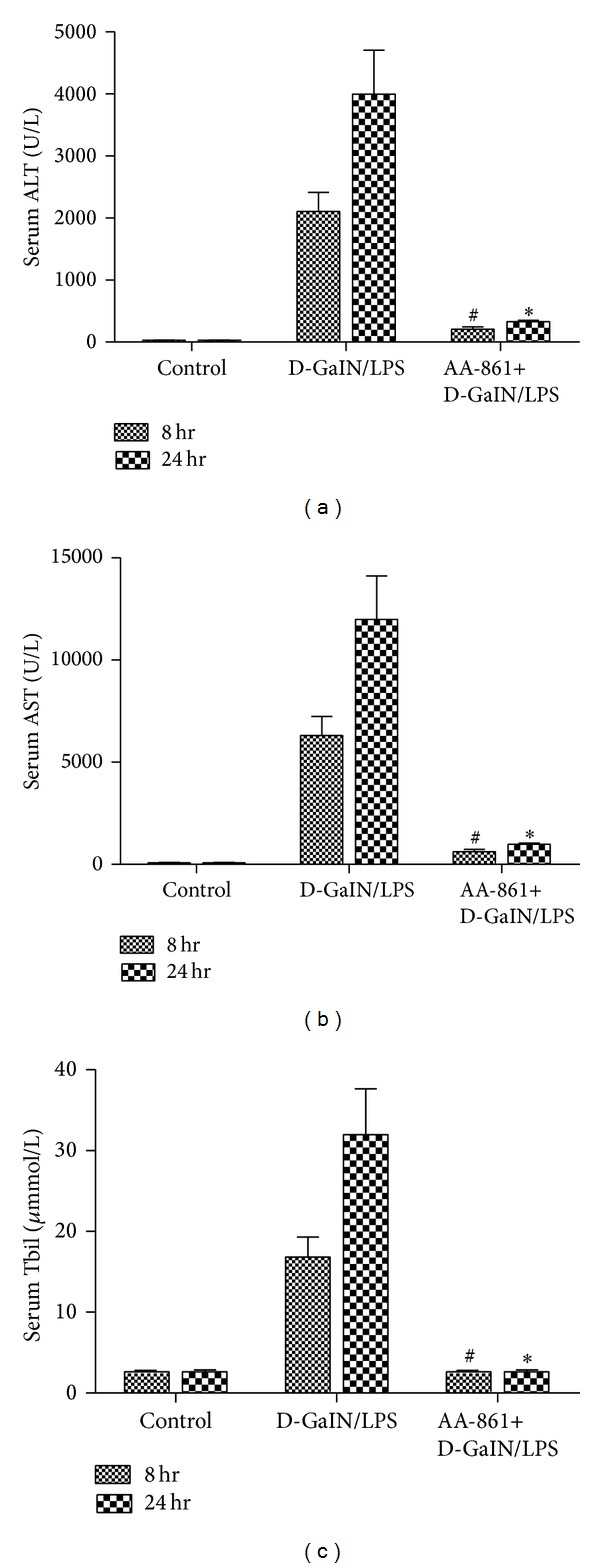
5-LO specific inhibitor AA-861 decreased levels of serum ALT, AST, and Tbil in D-GalN/LPS-treated rats. ((a)–(c)) Compared with experimental groups, the levels of serum ALT, AST, and Tbil in the AA-861 treatment groups were significantly decreased 8 and 24 hr after induction of ALF (*P* < 0.05).

**Figure 3:**
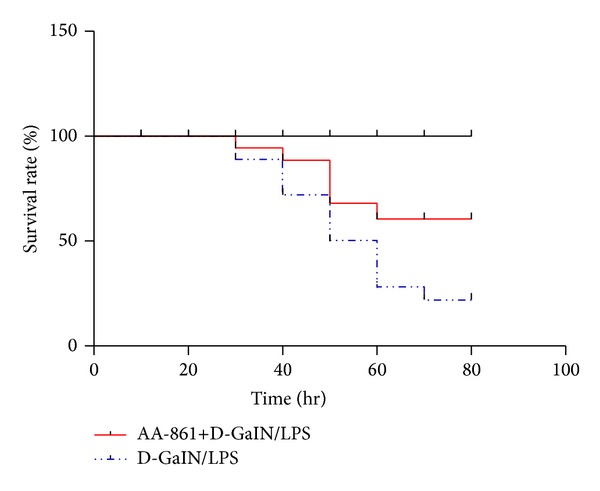
Blocking the 5-LO synthetic pathway improved the survival rate in D-GalN/LPS-treated animals. The survival rate in acute hepatic group and AA-861 treatment group.

D-GalN/LPS-treated rats showed massive hepatocyte necrosis at the centrilobular zone and bridging necrosis severely disrupted the sinusoidal and lobular architecture of the liver (necroinflammatory score; control versus D-GalN/LPS versus AA-861: 0.33 ± 0.21 versus 8.50 ± 0.22 versus 3.2 ± 0.3, *P* < 0.01; Figure 1). Serum ALT, AST, and Tbil levels of D-GalN/LPS-treated rats were significantly elevated compared with controls 8 hr after administering D-GalN/LPS (2104.3 ± 124.3 versus 27.3 ± 1 U/L, 5384.2 ± 335.8 versus 94.2 ± 3.2 U/L, and 19.7 ± 1.3 versus 2.2 ± 0.3 *μ*mmol/L, resp., Figure 2). These liver damage markers reached peak value 24 hr after administering D-GalN/LPS (3992 ± 290.7 U/L, 10114.7 ± 1014.2 U/L, and 32.9 ± 2.0 *μ*mmol/L, resp.). 5-LO inhibitor AA-861 significantly alleviated the hepatocellular necrosis (Figure 1) and the serum aminotransferases (Figure 2). Moreover, 80 hr after D-GalN/LPS injection, 14 rats were dead, and 13/20 rats survived in the AA-861-treated group (Figure 3).

**Figure 4:**
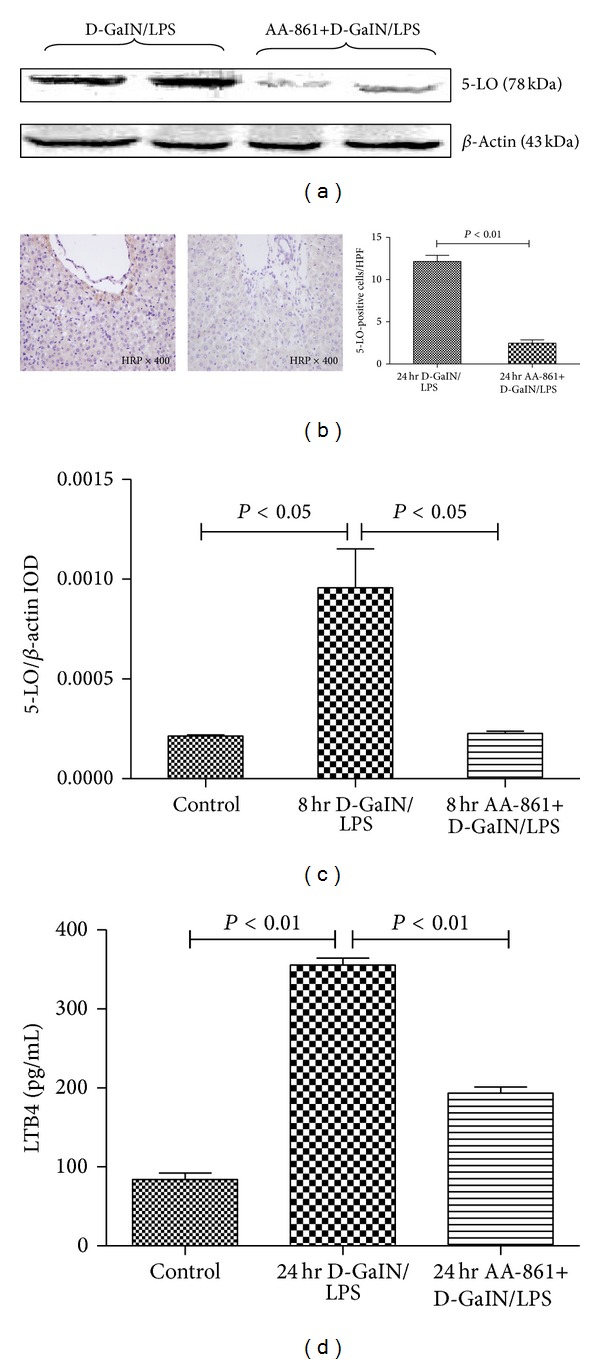
AA-861 significantly decreased total hepatic 5-LO protein and mRNA expression in D-GalN/LPS-treated animals. (a) Representative western blot analysis of the total 5-LO protein expression in liver samples 24 hr after D-GalN/LPS treatment in animals receiving placebo or AA-861. (b) AA-861 significantly abrogated the increased number of 5-LO-positive cells in the liver from D-GalN/LPS-treated rats. Representative photomicrographs (400x) of liver sections immunostained with 5-LO antibody 24 hr after D-GalN/LPS-treated rats received placebo (left panels) or AA-861 (right panels). (c) Representative RT-PCR analysis of 5-LO mRNA expression in liver samples 8 hr after D-GalN/LPS treatment in animals receiving placebo or AA-861. (d) AA-861 significantly reduced LTB4 levels in liver from D-GalN/LPS-treated rats. Liver tissue was obtained 24 hr after D-GalN/LPS treatment from animals receiving placebo or AA-861. Samples were homogenized and extracted in C18-silica, reverse-phase cartridges, and LTB4 was determined by highly specific enzyme immunoassay. Results represent the mean ± standard error.

Our previous study demonstrated that expression of total 5-LO protein reached its peak value 24 hr after administration of D-GalN/LPS, while the expression of 5-LO mRNA reached the highest level 8 hr [19]. AA-861 significantly decreased intrahepatic 5-LO total protein (Figure 4(a)) (5-LO-positive cells: 12.3 ± 0.7/HPF versus 2.5 ± 0.4/HPF, *P* < 0.01; Figure 4(b)) 24 hr after administration of D-GalN/LPS and 5-LO mRNA expression (Figure 4(c)) compared with the ALF group (5-LO/**β**-actin IOD: 9.6 × 10^−4^ ± 1.9 × 10^−4^  versus  2.3 × 10^−4^ ± 1.2 × 10^−5^,  *P* < 0.01) 8 hr after administration of D-GalN/LPS. In addition, 24 hr after D-GalN/LPS injection, LTB4, one of the 5-LO products, was significantly elevated in the liver tissue compared with controls (84.1 ± 8.1 versus 355.4 ± 22.2 pg/mL, *P* < 0.01; Figure 4(d)), while AA-861 treatment significantly decreased LTB4 (355.4 ± 22.2 versus 176.5 ± 18.5 pg/mL, *P* < 0.01; Figure 4(d)).

**Figure 5:**
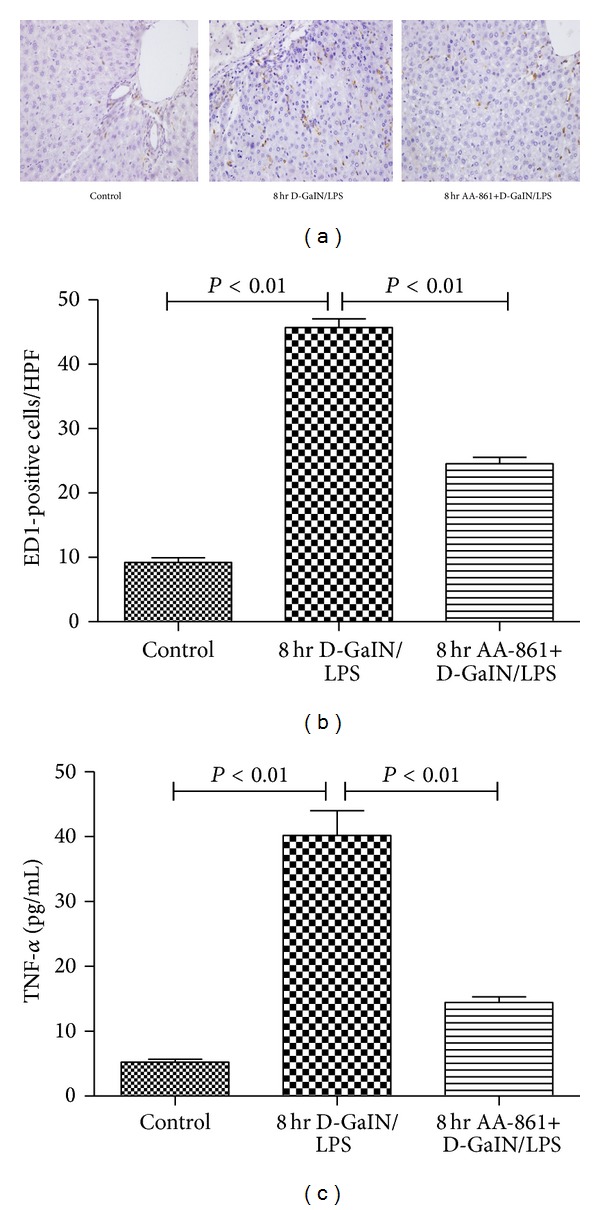
AA-861 significantly abrogated the increased number of ED1-positive cells in liver and increased level of TNF-*α* in serum from D-GalN/LPS-treated rats. (a) Representative photomicrographs (400x) of liver sections immunostained with ED1 antibody from control (upper left panel) and 8-hr D-GalN/LPS-treated rats receiving placebo (middle panel) or AA-861 (right panel). A representative number of ED1-positive cells (Kupffer cells) are denoted by arrows. In D-GalN/LPS-treated rats, ED1-positive cells were located mainly in the midzonal area surrounding the damaged perivenular area. (b) The number of ED1-positive cells per HPF in liver tissue sections from control (left bar) and D-GalN/LPS-treated animals receiving placebo (middle bar) or AA-861 (right bar) for 8 hr. (c) The level of TNF-*α* in serum from control (left bar) and D-GalN/LPS-treated animals receiving placebo (middle bar) or AA-861 (right bar) for 8 hr. Results are expressed as mean ± standard error.

In parallel with 5-LO, the macrophage density, stained with the ED1 monoclonal antibody, was significantly higher 8 hr after D-GalN/LPS administration (9.2 ± 0.7 versus 45.7 ± 1.3, *P* < 0.01; Figure 5(b)), and inhibition of 5-LO significantly decreased the macrophage density (24.6 ± 1.0 versus 45.7 ± 1.3, *P* < 0.01; Figure 5(b)) in liver tissue. TNF-*α*, mainly produced by macrophages, was significantly increased in D-GalN/LPS-treated rats compared with controls (40.2 ± 3.8 versus 5.2 ± 0.5  , *P* < 0.01; Figure 5(c)). AA-861 inhibited 5-LO, decreased macrophages in the liver of rats with D-GalN/LPS-induced ALF, and mitigated the increase in TNF-*α* concentration in the liver (40.2 ± 3.8 versus 14.4 ± 0.9, *P* < 0.01; Figure 5(c)).

**Figure 6:**
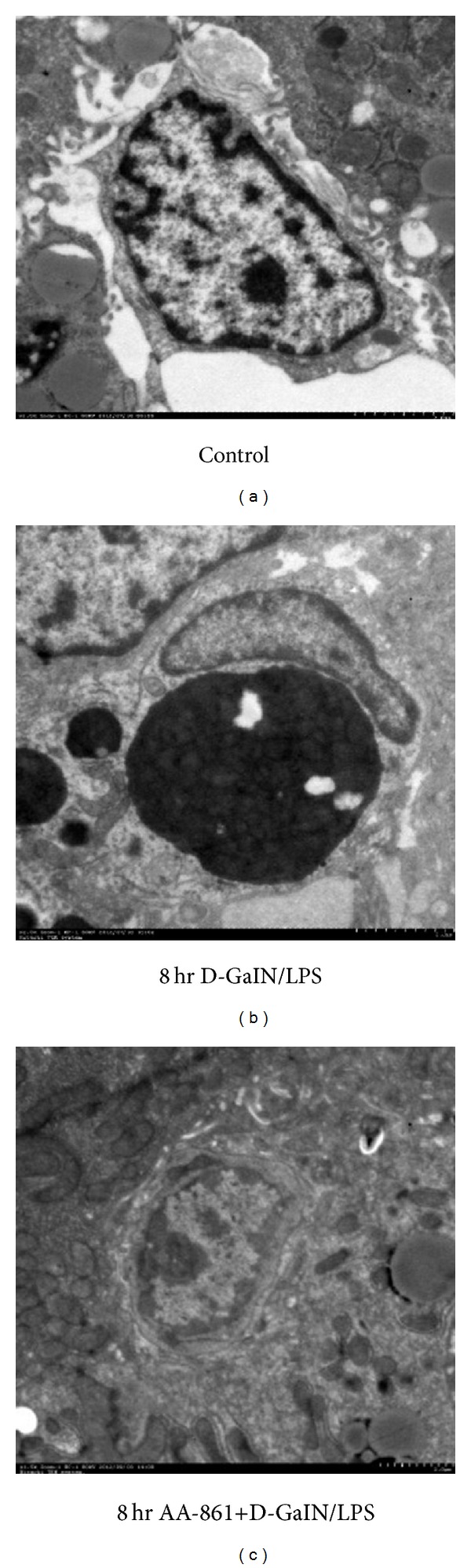
AA-861 markedly inhibited the morphological changes in macrophages. Representative electron micrographs (the scale for the picture is 2 *μ*m) of liver sections from control (a) and 8 hr D-GalN/LPS-treated rats receiving placebo (b) or AA-861 (c).

Transmission electron microscopy showed that the inactivated macrophages in liver tissue had an irregular round oval shape and small volume, the membrane surface had flat microvilli, and there were no obvious lysosomes or endoplasmic reticulum. The macrophages from the liver of rats with D-GalN/LPS-induced ALF showed a marked increase in size. Their outline was more regular and round, and the core of macrophages moved close to the membrane. The volume of chondriosomes increased significantly, and both lysosomes and ribosomal particles were seen, in addition to more microvilli and wrinkles on the cell surface. AA-861 treatment reversed the morphological changes in the macrophages, the cells were smaller, and the numbers of lysosomes and ribosomes in the cytoplasm were reduced (Figure 6).

## 4. Discussion

To the best of our knowledge, the present study is the first to show that macrophages play a critical role in D-GalN/LPS-induced ALF via the 5-LO pathway. It also showed that 5-LO inhibitor significantly alleviated D-GalN/LPS-induced ALF and that the mechanism of action may be related to inhibition of macrophage activation.

Peritoneal injection of D-GalN with LPS induces liver damage that closely resembles human viral hepatitis [20–22]. Thus, we chose to use this method to construct a rat model to imitate ALF caused by viral hepatitis in humans. We showed that D-GalN/LPS administration caused ALF, which was manifested as massive liver necrosis and significantly increased serum ALT, AST, and Tbil. Our previous study indicated that the 5-LO/LT pathway was involved in the initiation and progress of ALF induced by D-GalN/LPS [19]. So, in the present study, we investigated the relationship between 5-LO formation and macrophage activation. In addition, FLAP is also an integral component of the 5-LO pathway and its expression is crucial for the formation of LTs from arachidonic acid. However, the FLAP mRNA is not synthesized in macrophages specifically. Thus, we focused on 5-LO individually.

The first part of our study showed that AA-861 (a 5-LO inhibitor) alleviated the necroinflammatory damage to the liver in rats induced by D-GalN/LPS and improved the survival rate. From other researches, we selected the dose of AA-861 as 20–60 mg/kg [23, 24]. Thus, before the present study, we used the dose of 30 mg/kg and 60 mg/kg to do the previous experiment, respectively, and the results showed that a dose of 60 mg/kg was better. The present study proved that the 5-LO/LT pathway was involved in the progress of ALF induced by D-GalN/LPS. Blocking this pathway reduced the severity of the damage in ALF, which may offer us a new approach in the treatment of ALF by inhibition of this pathway in the future. The level of the main metabolite of the 5-LO pathway, LTB4, a potent chemotactic agent for leukocytes [25], was decreased. Our results also confirmed that 5-LO products, as mediators of inflammation and cell damage, play important roles in the pathogenesis of hepatocellular injury [26–30]. The protective effects of AA-861 on ALF were mediated by downregulation of 5-LO and its products.

It is well known that macrophages are one of the major cell types involved in initiating specific immune responses [31]. In ALF, macrophages take part in the process of the “first hit” and play an important role in the process of the “second hit” [32]. Some researchers have found that the expression of 5-LO in the liver is restricted to macrophages [15]. So, the second part of our study focused on the relationship between 5-LO formation and macrophage activation.

Using transmission electron microscopy, our previous study [33] showed the morphological changes in the macrophages in D-GalN/LPS-induced inflammation. We found that, 8 hr after D-GalN/LPS injection, the microstructural changes in the macrophages were obvious. The phagocytic and secretion activity also reached peak levels. The expression of 5-LO mRNA in liver tissue also reached a peak 8 hr after administration of D-GalN/LPS; therefore, we assumed that the synthesis of 5-LO is related to macrophage activation.

To further evaluate the potential relationship between synthesis of 5-LO and macrophage activation in the progression of D-GalN/LPS-induced ALF, we took AA-861 and found that it inhibited 5-LO formation in partially depleted ED1-positive cells (macrophages). The large increase in the number of ED1-positive cells reflected macrophage activation; therefore, we concluded that blocking the activity of 5-LO impeded macrophage activation. In addition, the prevention of hepatic necroinflammation may be associated with depletion of macrophages, which are increased in response to D-GalN/LPS injection. Some studies have demonstrated that macrophage depletion can alleviate the inflammatory response, such as endotoxin-induced lung inflammation [34] and CCl_4_-induced liver injury [14]. Our results are in line with other studies in other organisms.

In addition, the anti-inflammatory effect of AA-861 on D-GalN/LPS-induced ALF in rats was also mediated through inhibition of TNF-*α*, which is mainly produced by macrophages and considered to be one of the main cytokines involved in hepatocellular damage [35]. Moreover, 5-LO inhibition changed the microstructure of the macrophages, which included the size of macrophages and the number of lysosomes and ribosomes in the cytoplasm. All of these findings implied that blocking the activity of 5-LO downregulated macrophage proliferation and inhibited macrophages' function.

In conclusion, 5-LO inhibition decreased the density of macrophages and proinflammatory cytokines and alleviated D-GalN/LPS-induced liver damage. These data may help us design further studies in the treatment of ALF via inhibition of macrophage activation in the future.
